# CCK reduces the food intake mainly through CCK1R in Siberian sturgeon (*Acipenser baerii* Brandt)

**DOI:** 10.1038/s41598-017-12646-3

**Published:** 2017-09-29

**Authors:** Xin Zhang, Ni Tang, Jinwen Qi, Shuyao Wang, Jin Hao, Yuanbing Wu, Hu Chen, Zhengzhi Tian, Bin Wang, Defang Chen, Zhiqiong Li

**Affiliations:** Department of Aquaculture, College of Animal Science and Technology, Sichuan Agricultural University, 211# Huimin Road, Chengdu, China

## Abstract

To explore the effect of CCK on food intake in Siberian sturgeon, *cck* cDNA sequence of 1005 bp was obtained, and *cck* mRNA possessed the highest expression in brain. The expressions of *cck* were significantly increased after feeding 1 and 3 h, while displaying significant decrease after fasting within 15 days in brain and duodenum. Re-feeding for 3 days induced *cck* level returned to basic level. Acute *i*.*p*. injection experiment showed 100 and 200 ng/g BW CCK8 inhibited the food intake in 0–1 h together with the cumulative food intake within 3 h. 7 days chronic *i*.*p*. injection of 100 and 200 ng/g BW CCK8, both daily food intake and cumulative food intake were significantly decreased. In addition, chronic *i*.*p* injection of CCK8 induced the expression of feeding related factors changes including *cck*, *ucn3*, *cart*, *apelin*, *pyy* and *npy* in respective organization. Moreover, as revealed by the results, Lorglumide, the CCK1R selective antagonist, effectively reversed the inhibitory effects of CCK8 on food intake and the levels of feeding related factors. On the other hand, LY 225910, the CCK2R selective antagonist, partially reversed these effects. These results indicate CCK is a satiety factor inhibits the feeding of Siberian sturgeon primarily through CCK1R.

## Introduction

Feeding, a complex behavior, is essential to promote animal survival and might be affected by factors, such as light, temperature, reproduction and even the type of food consumed. Food intake is controlled by a central and peripheral feeding system connected by a network of peptides and hormones that regulate hunger and satiety^1–3^. On this intricate network, an intestinal peptide named CCK, also detected in the brain, has been recently implicated on feeding regulation in rats^4,5^.

The role of CCK on feeding was studied on a variety of animals. In teleost, *cck* has been cloned in goldfish^6^, Japanese flounder^7^, yellowtail^8^, Atlantic salmon^9^, winter skate^10^, red drum^11^, grass carp^12^, mandarin fish^13^, *Schizothorax prenant*i^14^ and blunt snout bream^15^, but there is no information about CCK in *Acipenseridae* species. It has been discovered that CCK is capable of inhibiting the animal feeding through the central and peripheral systems^16^, while the role of CCK in feeding is still limited in teleost.

Siberian sturgeon (*Acipenser baerii* Brandt), a kind of sub-cold water fish^17^ is frequently farmed across the globe. However, few studies focused on Siberian sturgeon appetite regulation, such as PYY^18^, UCN3^19^ and Apelin^20^. Recently, our laboratory acquired the responsibility of delivering a better understanding of the appetite regulation in Siberian sturgeon through the study of appetite factors. The role of feeding regulators and their interaction is an issue that requires immediate investigation.

To investigate the feeding regulation of CCK in *Acipenseridae* species, the cDNA sequence of *cck* was cloned for the first time and its tissue distribution was detected. Thereafter, the short-term and long-term feeding effects of CCK were studied by *periprandial* (pre- and post-feeding) experiments, fasting and re-feeding experiments, as well as acute and chronic *i*.*p*. injection experiments. Ultimately, the CCK8, together with its receptor selective antagonists co-injection experiments were utilized for the detection of the amount of food intake and the expression of feeding related factors, with an aim of studying the mechanism of CCK in feeding regulation.

## Materials and Methods

### Fish

Siberian sturgeons were purchased by Runzhao Fisheries Co., Ltd. (Sichuan, China). 294 fishes were kept in 60.0 × 50.0 × 40.0 cm^3^ indoor tanks and under the natural photoperiod (12 h light: 12 h dark) in Sichuan Agricultural University Aquaculture Laboratory (Chengdu, China). All tanks were consistently aerated, in addition to the supply of fresh water at 20 ± 1 °C. Fishes were fed to satiety once-daily at 14:00, with commercial sinking pellets (crude protein ≥40%, crude fat ≥12%, coarse fiber ≤6%, crude ash ≤18%; Tongyi, Suzhou, China). Fishes were acclimated subjected to these conditions for a period of two weeks prior to the start of the experiments. We chose the circulating water system. In addition, the wastes and uneaten pellets were also collected. Fishes were anesthetized in 0.02% tricaine methane sulfonate (MS-222) before intraperitoneal (*i*.*p*.) injection or sampling. All the experiments were accordance with the Animal Care and Use Committee of Sichuan Agricultural University, under permit No.DKY-S20150812.

### Tissues sampling, cloning and sequence analysis

In respect of cloning and tissue distribution, Siberian sturgeons (438.77 ± 59.72 g, n = 6, indoor tank with a diameter of 95 cm and a height of 85 cm) were sampled at 1 h after feeding time. 11 feeding-related tissues were sampled^21^, including whole brain, esophagus, stomach, pyloric caeca, duodenum, valvula intestine, rectum, liver, pancreas, spleen and trunk kidney. All the tissues were washed with sterile saline, snap-frozen in liquid nitrogen, and stored at −80 °C until total RNA isolation.

Total RNA from Siberian sturgeon brain was isolated with the use of the RNAiso Plus (Takara, Dalian, China) and cDNA synthesis using the PrimeScript™ RT Reagent Kit (Takara, Dalian, China) in accordance with the manufacturer’s protocol. The integrity of total RNA was measured by electrophoresis, and then the optical density absorption ratios at wavelengths of 260 nm and 280 nm (A260/280) were determined by a photometer (Bio-Rad, USA). The high-quantity RNAs (A260/280 > 1.8) used for cDNA synthesis. First-strand cDNA of whole brain with 5′ or 3′ adaptors added was synthesized using SMART RACE cDNA Construction Kit (Clontech, USA) for 5′ and 3′ rapid amplification of cDNA ends (RACE). PCR products were purified from 1.5% agarose gel by Universal DNA Purification Kit (TIANGEN, Beijing China), ligated into the pMD-19T vector (TaKaRa, Dalian, China), and then introduced in the competent cells *E*. *coli* DH5α (Takara, Dalian, China). Sequencing was performed at Beijing Genomics Institute (BGI, Chongqing, China). The coding sequence cloning primers were designed in accordance with the Siberian sturgeon whole brain transcriptome data (unpublished) as well as other vertebrates *cck* genes. Cloning of 5′ and 3′ RACE primers were designed using Primer premier 5.0 program and are listed in Table 1 (*cck*-R, *cck*-F; *cck*-5′ R1, *cck*-5′ R2; *cck*-3′ F1, *cck*-3′ F2). The PCR conditions used were described as previously^22^.

**Table 1:** Primer sequences and function used in this study.

Primer name	Primer sequence (5′ to 3′)	Applications
*cck*-F	GCTATGAACAGTGGAATC	*cck*-cloning
*cck*-R	GGCTACCCACTGTATTGT	*cck*-cloning
*cck*-F1	TGAAAACGGCAGAGGGAAACAT	*cck*-3′ RACE outer
*cck*-F2	TTTAGAGCCAGACAGTTGGGAGA	*cck*-3′ RACE inner
*cck*-R1	CGTCCAAAGTCCATCCAGCCCAT	*cck*-5′ RACE outer
*cck*-R2	GTCCAAGGTTCGCTCTTTGCTCC	*cck*-5′ RACE inner
*cck*-qF	GAGGGTAGTCCTGTAGCATCTGA	*cck*-qPCR
*cck*-qR	TTCTACCAGACGAGCCTTTCC	*cck*-qPCR
*ucn3*-qF	CAGGGGAGGAGAGAGAAAAAA	*ucn3*-qPCR
*ucn3*-qR	CTGAGACATTAGGCGAGCGT	*ucn3*-qPCR
*nucb2*-qF	TGGAGACAGACCAGCATTTCAG	*nucb2*-qPCR
*nucb2*-qR	GGCTCCGTAACCTGTTCACTTC	*nucb2*-qPCR
*cart*-qF	CGACTGTGGTTGAGAGCCG	*cart*-qPCR
*cart*-qR	GACAGTCACACAACTTGCCGAT	*cart*-qPCR
*apelin*-qF	CAGACACGCTGTTTTACACCAC	*apelin*-qPCR
*apelin*-qR	GCACAGCATGGACACCAAGAT	*apelin*-qPCR
*pyy*-qF	AGGCAGAGGTATGGCAAGCG	*pyy*-qPCR
*pyy*-qR	GGAGGGTCAGGAGACGGGAT	*pyy*-qPCR
*npy*-qF	GCTGGCTACCGTGGCTTTC	*npy*-qPCR
*npy*-qR	GACTGGACCTCTTCCCATACCT	*npy*-qPCR
*β*-*actin*-qF	GTTGGTATGGGACAGAAGGACA	*β*-*actin*-qPCR
*β*-*actin*-qR	CCAGTTGGTAACAATGCCGT	*β*-*actin*-qPCR
*gapdh*-qF	CATTTGATGTTGGCTGGGT	*gapdh*-qPCR
*gapdh*-qR	CTTTCTGGGAAGGTGGAGGT	*gapdh*-qPCR

The *cck* cDNA and deduced amino acid sequences were analyzed by employing BLASTn and BLASTp (http://www.ncbi.nlm.nih.gov). The *cck* ORF was predicted with the Open Reading Frame Finder (http://www.ncbi.nlm.nih.gov/gorf/gorf.html). Besides, the cleavage site of the signal peptide was predicted by using SignalP 4.1 Server (http://www.cbs.dtu.dk/services/SignalP/). Furthermore, multiple alignments of amino acid sequences and phylogenetic analysis were performed using MEGA 7.0 software (http://www.megasoftware.net/index.html). The phylogenetic tree was constructed by the maximum likelihood method. The analysis reliability was assessed by 1000 bootstrap replicates.

### Periprandial, fasting and re-feeding experiments

For *periprandial* (pre- and post-feeding) experiments, 63 fish (29.46 ± 3.56 g) were randomly distributed among 7 tanks (9 fish per tank). No differents feeding behavior were observed among these tanks prior to the experiment. All through the experiment, 5 tanks were fed with normal feeding procedure, whereas other 2 tanks were fasted at the feeding point (14:00). At sampled points, random selection of 6 fish in each tank was made for sampling purpose. Sampling of the whole brain and duodenum of fish in 5 tanks was performed at 3 h (11:00, −3 h) and 1 h (13:00, −1 h) prior to feeding, at start feeding (14:00, 0 h), 1 h (15:00, +1 h) and 3 h (17:00, +3 h) after feeding, correspondingly. Other 2 fasting tanks were sampled at +1 h and +3 h as the control of the feeding fish. The whole brain and duodenum were sampled, together with numbering and flash freezing in liquid nitrogen and storage at −80 °C, for subsequent use in RNA isolation.

For the fasting and re-feeding experiments, random distribution of 117 fish (29.45 ± 2.84 g) was performed among 13 tanks (9 fish per tank) including 6 tanks of fasting, 5 tanks of feeding and 2 tanks of re-feeding subsequent to 15 days of fasting. Fasting fish were not fed at 14:00 at the experiment day. Furthermore, 6 individuals were randomly sampled from each tank at the following time points: 1, 3, 6, 10, 15 days feeding; 1, 3, 6, 10, 15, 17 days fasting; 15th day (re-feeding 1 day) and 17th day (re-feeding 3 days) re-feeding. Both the whole brain and the duodenum were collected as mentioned above.

### Peptide and drugs

Siberian sturgeon sulfated cholecystokinin octapeptide (DY^-SO3H^MGWMDF, CCK8) was custom synthesized with purity of 95.13% by Shanghai Bootech BioScience &Technology Co.,Ltd.(Shanghai, China). The purity was verified through the application of High Performance Liquid Chromatography (HPLC) analysis. Peptide was dissolved in fish physiological saline for 0, 50, 100 and 200 ng·μl^−1^. Doses were selected on the bases of the report of Penney and Volkoff^23^. Lorglumide (4-[(3, 4-dichlorobenzoyl) amino]-5-(dipentylamino)-5-oxo- pentanoic acid sodium salt; Cayman chemical; USA) is a CCK 1 receptor antagonist. It was dissolved in fish physiological saline for 1 μg·μl^−1^ which was referenced to Hayes *et al*.^24^. LY 225910 (2-[2-(5-Bromo-1 H-indol-3-yl) ethyl]-3-[3-(1- methylethoxyphenyl]-4(3 H)–quinazolinone; Abcam, USA) is a CCK 2 receptor antagonist. It was dissolved in fish physiological saline with a few drops of dimethyl sulphoxide and then saline was added to 0.5 μg·μl^−1^ which was referenced to Ballaz *et al*.^25^. All the drugs were stored at −20 °C until intraperitoneal (*i*.*p*.) injection experiments.

### Effect of i.p. injection of CCK8 on food intake and sampling

The investigations were carried out with acute and chronic *i*.*p*. injection of CCK8, for examining whether the exogenous CCK exerts any impact on Siberian sturgeon food intake. In respect of the acute administration of CCK8, weight-matched 36 fish (121.90 ± 10.38 g) were randomly divided into four groups (12 tanks, 3 fish per tank). Daily feeding of fish was performed and conditioned for two weeks. Since an acute study was performed, the fish were required to adapt to the anesthetized and *i*.*p*. injected procedure. Two days before the experimental day, fishes were anesthetized with MS-222, and *i*.*p*. administrated with saline at 13:30 (30 min for fish returning to the normal state prior to feeding). On the day of the experiment, weights of fish were recorded, and *i*.*p*. injection of the CCK8 solution with 0, 50, 100 and 200 ng/g BW for four groups, respectively. The experiment was carried out at the feeding time (14:00, 0 h) when the fish had adapted to eat, and there was no difference in food intake. Each fish was fed with the pre-weighed sinking pellets diet in excess of 3% body weight ratio at 0 h, 1 h and 3 h. Residual diets were collected at 1 h, 3 h and 6 h, and weighed after drying for determining the food intake. Simply said, during the acclimation period, pre-weighted feed was immersed in water oxygenated for the simulation of fish swimming for 1 h, 2 h or 3 h, correspondingly. Thereafter, collection of the feed was done, together with drying and weighing for the purpose of calculating the proportion of the weight to the fresh weight. In accordance with the proportion, the fresh weight of the remaining feed was calculated in the feeding experiments, and the fresh weight of feed intake by fish was attained through the subtraction of the fresh of the remaining feed from the initial feeding amount. In the long run, in accordance with the feed intake of fresh weight than fish total weight, food intake was recorded as mg/g BW.

As regarding the chronic administration of CCK8, 36 weight-matched fishes (119.43 ± 8.51 g) were randomly divided into four groups (12 tanks, 3 fish per tank). The fish were conditioned for 2 weeks. Fish were given daily *i*.*p*. administration of CCK8 for 7 days with 0, 50, 100 and 200 ng/g BW for four groups, correspondingly whereby the procedures were the same as stated above. Fish were fed with the pre-weighed diet at 0 h, followed by the collection of uneaten pellets at 1 h to measure 1 h food intake each day. To exploring the regulation mechanism of CCK8 continuous injection on food intake, sampling of the brain, stomach and duodenum was carried out at 1 h on the day 7 as stated in the section 2.3.

### Effect of i.p. co-injection of CCK8 and CCK receptor antagonists on food intake and sampling

In order to investigate the mechanism of CCK8 inhibiting the food intake, *i*.*p*. co-injection of CCK8 and its receptor antagonists (Lorglumide and LY 225910) were performed. Random division of 36 weight-matched fish (162.54 ± 20.03 g) was performed into four groups (12 tanks, 3 fish per tank). Fish were fed every day. The fish were acclimated for two weeks. Since injection procedure was performed, the fish were required to adaptive procedures. On the day of the experiment, 6 tanks’ fishes were weighed and *i*.*p*. injection of Lorglumide (3 tanks, 1 μg·μl^−1^) and LY 225910 (3 tanks, 0.5 μg·μl^−1^) solution at 13:20, respectively. Then, these fishes were *i*.*p*. injected with CCK8 (100 ng/g BW) at 13:35^26^. Other 6 tanks’ fish were weighed and *i*.*p*. injected with CCK8 (3 tanks, 100 ng/g BW) and saline at 13:30, correspondingly. Each fish was fed with the pre-weighed sinking pellets diet being an excess of 3% body weight ratio at 0 h. Collection of the residual diets was performed at 1 h and weighed after drying for the determination of food intake. Sampling of the brain, stomach and duodenum was performed at 1 h as described in section 2.3.

### Real-time PCR analyses

Detection of the expressions of genes was performed by real-time quantitative PCR (qPCR), and the primers have presented in Table 1. We used both *β-actin* (Primers: *β-actin*-F, *β-actin*-R; Table 1) and *gapdh* (Primers: *gapdh*-F, *gapdh*-R; Table 1) as reference genes for the purpose of controlling for error between samples to analyze the target genes mRNA expressions. Procedures and methods were described as the previous way^22^. For all standard curves, the primer amplification efficiencies of genes were 96.3-99.5% and 0.971 < R^2^ < 0.997 respectively for all standard curves. The target genes were normalized to the reference genes (geometric averaging of *β-actin* and *gapdh* Ct value) and expression levels were compared with the relative Ct method^27^.

### Statistical analyses

All data were expressed as mean ± SEM. Statistical analyses were performed using SPSS (version 21.0) statistical software (SPSS Inc., Chicago, IL, USA). Student’s *t*-tests were used for the comparison between two groups. In respect of multiple group designs, subsequent to the confirmation of normal distribution of data, one-way ANOVA analysis was put to use followed by *LSD* post-hoc test. *P* < 0.05 was considered to be statistically significant.

## Results

### Molecular cloning and Phylogeny of Siberian sturgeon cck

**Figure 1:**
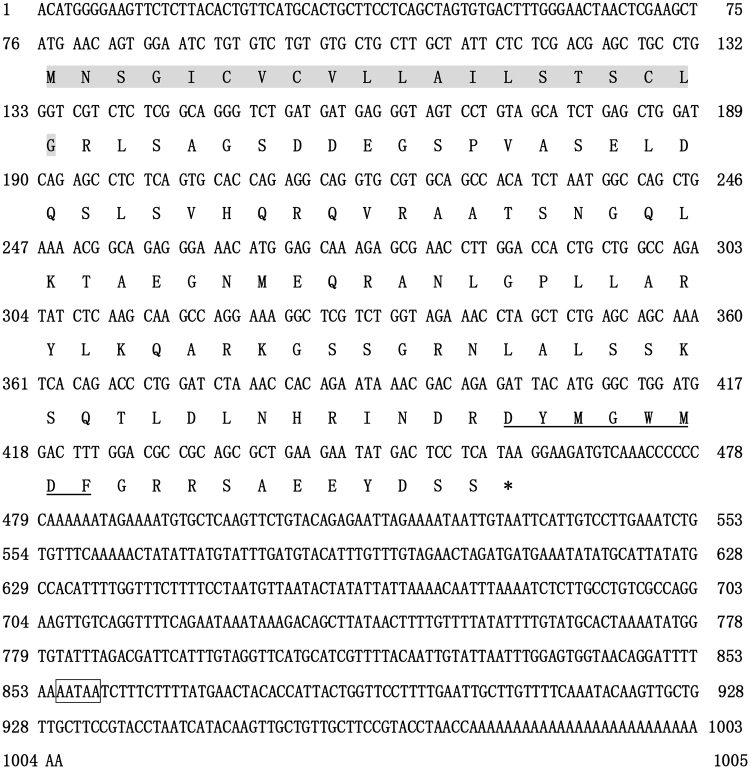
Nucleotide and deduced amino acid sequences for Siberian sturgeon (*Acipenser baerii*) *cck*. The putative signal peptide is shaded and cholecystokinin octapeptide is underlined. The asterisk indicates the stop codon.

Full-length cDNA sequence of Siberian sturgeon *cck* was obtained using RACE PCR. The *cck* nucleotide sequence was 1005 base pairs (bp) including a 384 bp open reading frame (ORF), a 75 bp 5′ untranslated region (5′ UTR) and a 546 bp 3′ untranslated region (3′ UTR). Siberian sturgeon *cck* ORF encoded a putative 127 amino acids precursor protein having a putative 20 amino acids signal peptide as well as the C-terminal octapeptide (CCK8) (Fig. 1). The part of CCK octapeptide was highly conserved between all species (Supplementary Fig. 1). However, the whole amino acid sequence of Siberian sturgeon CCK exhibited 64.6% identity with *Latimeria chalumnae* CCK and *Gallus gallus* CCK, as well as 40.9% with Rainbow trout CCK-L (Supplementary Fig. 1). Phylogenetic analysis showed that Siberian sturgeon CCK was clustered with *Latimeria chalumnae* CCK, while divided from other fish species’ CCK (Supplementary Fig. 2).

### Tissue distruction of Siberian sturgeon cck

**Figure 2:**
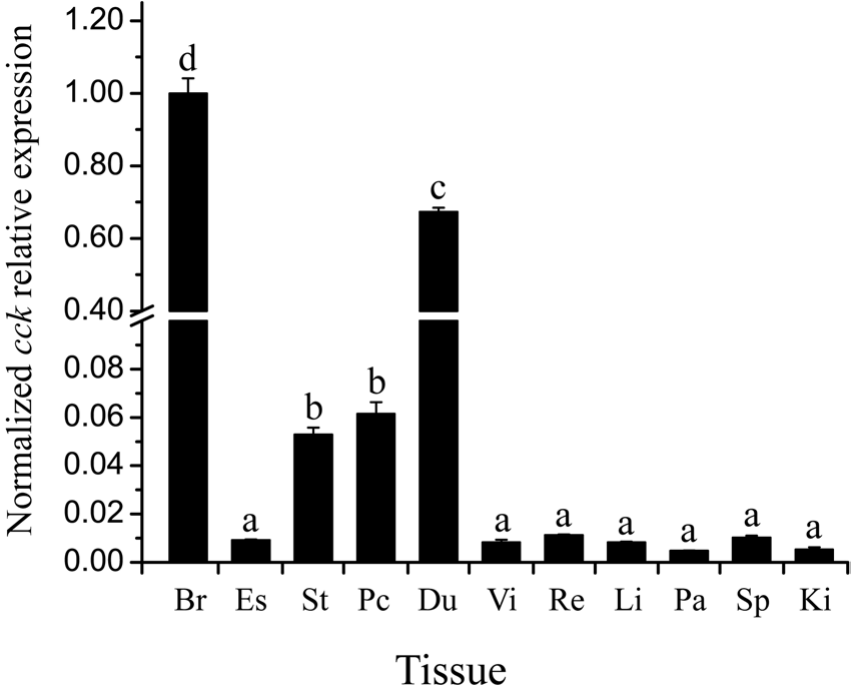
Tissue distribution of *cck* mRNA in Siberian sturgeon. The results were expressed as relative expression levels after standardization by *β-actin* and *gapdh*. Error bars represent standard error of the mean. Br, whole brain; Es, esophagus; St, stomach; Pc, pyloric caeca; Du, duodenum; Vi, valvula intestine; Re, rectum; Li, liver; Pa, pancreas; Sp, spleen and Ki, trunk kidney. Data are means ± SEM; n = 6 per tissue.

The *cck* mRNA was widely distributed in all tested tissues. The highest mRNA level of *cck* was observed in the whole brain, followed by duodenum, stomach and pyloric caeca, and low levels in other tissues (Fig. 2).

### Periprandial, fasting and re-feeding induced changes of cck mRNA in Siberian sturgeon brain and duodenum

**Figure 3:**
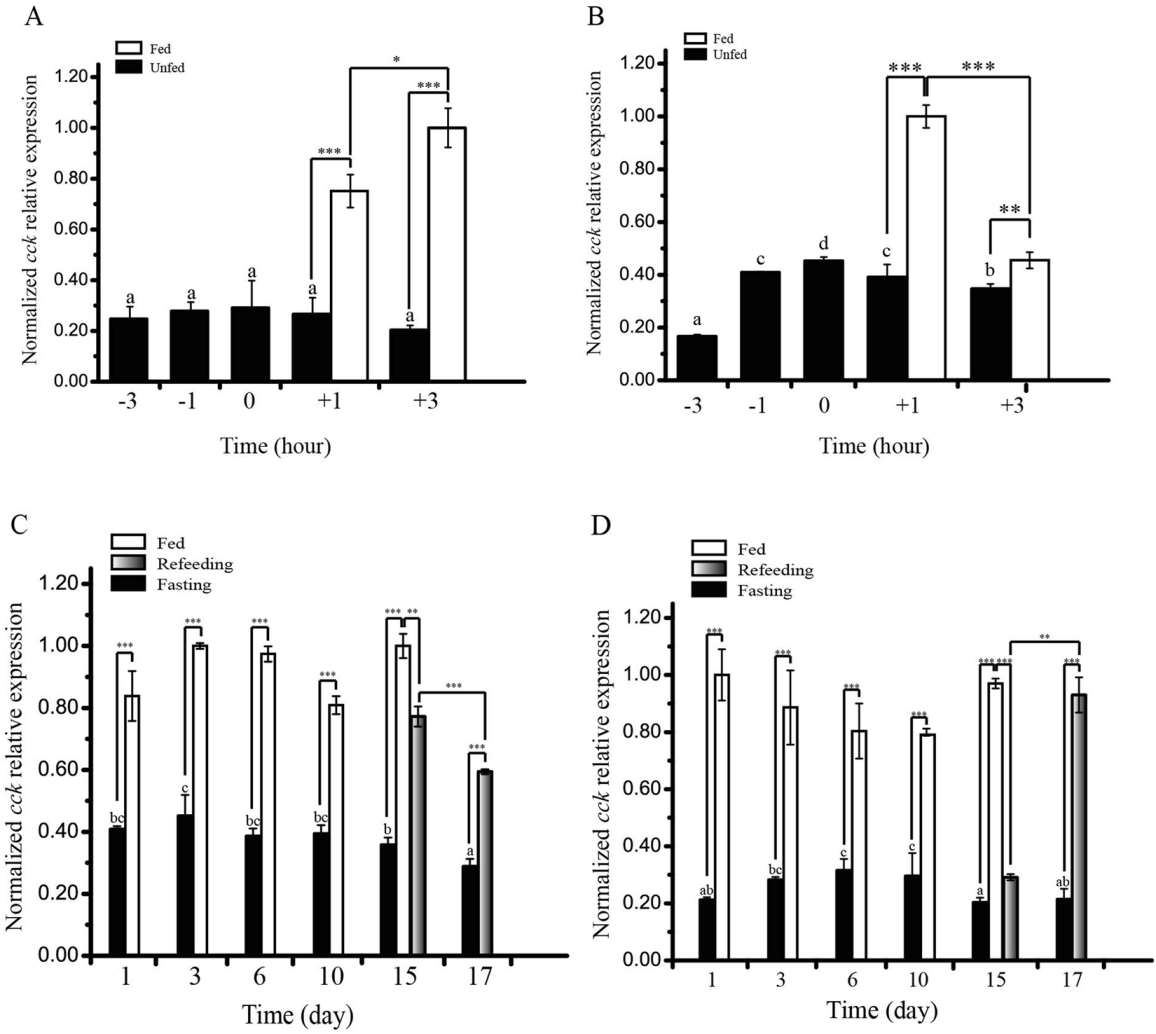
*Periprandial* experiments, fasting and re-feeding experiments induced changes in *cck* mRNA (**A**,**C**) are whole brain; (**B**,**D**) are duodenum). The mRNA expression was normalized to *β-actin* and *gapdh* genes, and the highest level of target gene mRNA expression was set equal to 1.0. Data are means ± SEM; n = 6 per group. Bars with different letters represent significant differences between unfed groups (ANOVA, *P* < 0.05). Asterisks represent significant differences between the two objects (Student’s *t*-test, *P < 0.05, **P < 0.01 and ***P < 0.001).

The findings obtained from the *periprandial* experiment revealed the fact that postprandial +1 h and +3 h *cck* expression levels in the whole brain and duodenum were significantly higher in comparison with those of unfed groups (Fig. 3A,B). Postprandial +3 h *cck* mRNA expression was also considerably higher as compared with that at +1 h in the whole brain, while it amounted to be lower than +1 h in duodenum (Fig. 3A,B).

As revealed by the findings, the *cck* mRNA expression possessed considerably decreased in fasting fish at 1, 3, 6, 10 and 15-day comparison with ad libitum fed fish (the basic level) in the whole brain and duodenum (Fig. 3C,D). When fish was re-fed subsequent to 15 days of fasting, the *cck* mRNA expression was significantly increased as compared with the 15-day ad libitum fed fish (Fig. 3C,D). Re-feeding for 3 days stimulated the *cck* mRNA level that considerably boosted corresponding with 17-day fasting fish in the whole brain, together with returning to the basic level, whereas, that amounted to be substantially lower than re-feeding for 1-day fish (Fig. 3C). However, the expression of *cck* mRNA in the duodenum was significantly increased corresponding to 17 days fasting fish and re-feeding for 1-day fish, and that was returned to the basic level, as well (Fig. 3D).

### Acute effects of i.p. injection of CCK8 on food intake

**Figure 4:**
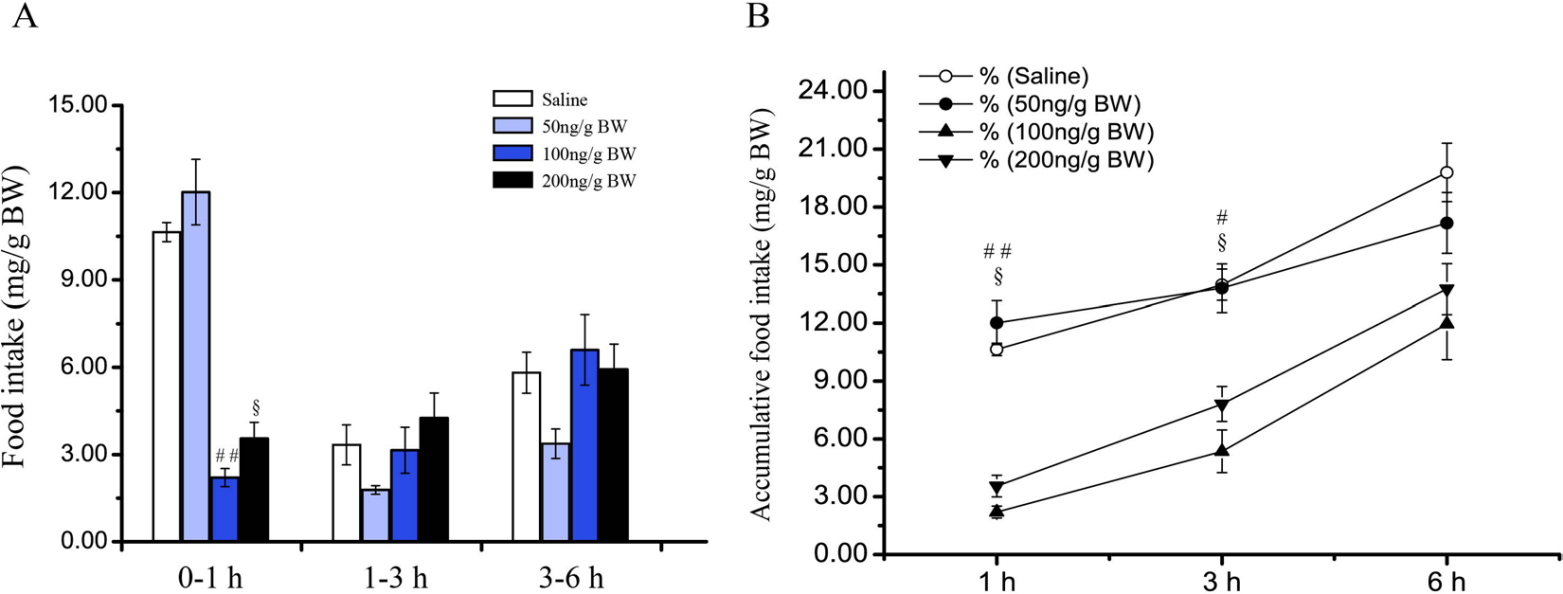
Effects of acute *i*.*p*. administration of CCK8 (50, 100 and 200 ng/g BW) on food intake by period time (**A**) and cumulative food intake (**B**) in Siberian sturgeon. Data are means ± SEM; n = 3 per group. Marks with dissimilar superscripts indicate groups that differ significantly by Student’s *t*-test. *P < 0.05 as 50 ng/g BW vs. the saline treated controls; ^#^ *P* < 0.05 and ^##^ *P* < 0.01 as 100 ng/g BW vs. the saline treated controls; ^§^ *P* < 0.05 as 200 ng/g BW vs. the saline treated controls.

For the purpose of investigating whether the exogenous CCK8 exerts any impact on Siberian sturgeon short-term feeding behavior, this study performed the examination of the effects of *i*.*p*. injection of 50, 100 and 200 ng/g BW CCK8 on 0–1, 1–3 and 3–6 h food intake of Siberian sturgeon. As revealed by the results, 100 and 200 ng/g BW CCK8 significantly reduced the food intake of fish in 0–1 h (100 ng/g BW, *P* < 0.05; 200 ng/g BW, *P* < 0.01. Figure 4A). Nonetheless, in 1–3 and 3–6 h CCK8 delivered no impact on food intake in relation to the saline group. In addition, the results demonstrated that CCK8 (100 and 200 ng/g BW) significantly reduced the cumulative food intake in 3 h (*P* < 0.05, Fig. 4B), but all three doses of CCK8 made no impact on the cumulative food intake in 6 h (Fig. 4B).

### Chronic effects of i.p. injection of CCK8 on food intake

**Figure 5:**
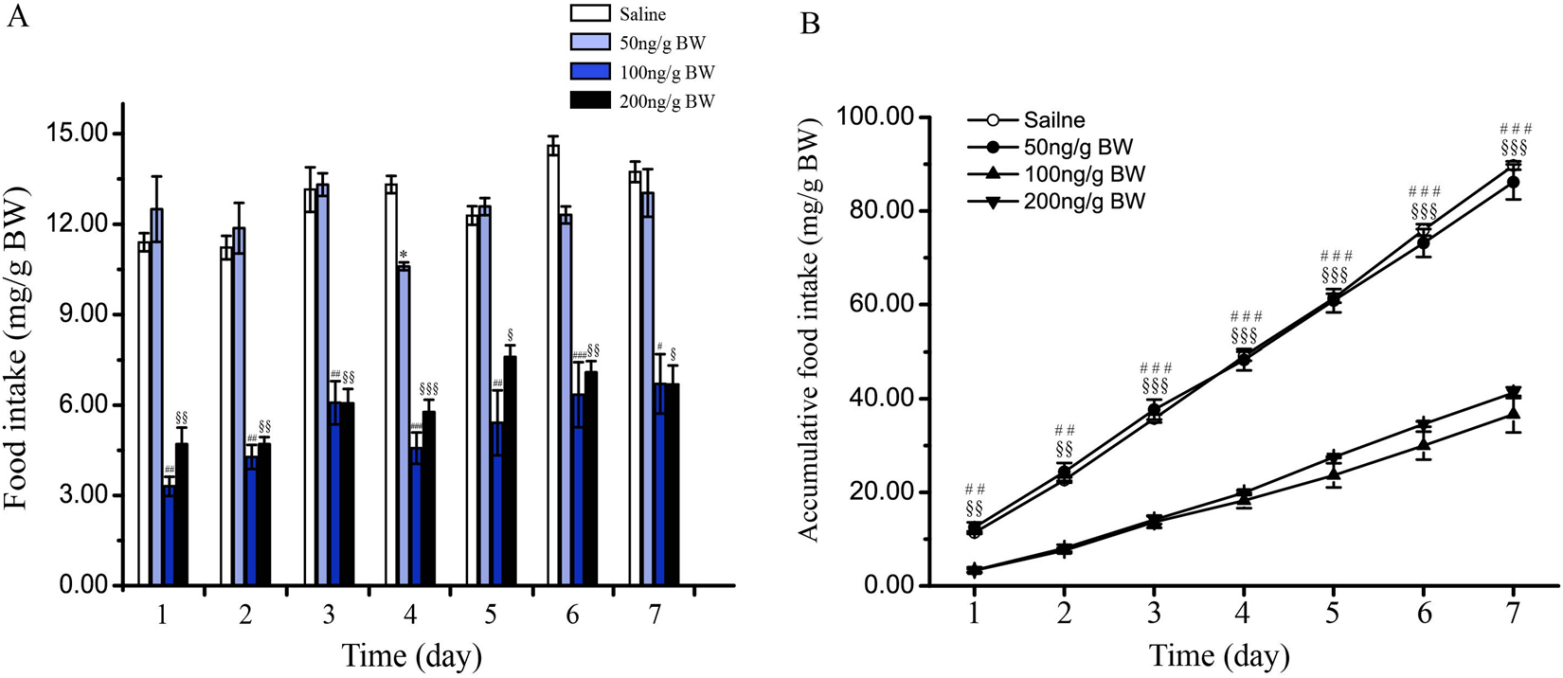
Effects of chronic *i*.*p*. administration of CCK8 (50, 100 and 200 ng/g BW) on food intake by period time (**A**) and cumulative food intake (**B**) in Siberian sturgeon for 7 days. Data are means ± SEM; n = 3 per group. Marks with dissimilar superscripts indicate groups that differ significantly by Student’s *t*-test. *P < 0.05 as 50 ng/g BW vs. the saline treated controls; ^#^ *P* < 0.05, ^##^ *P* < 0.01 and ^###^ *P* < 0.001 as 100 ng/g BW vs. the saline treated controls; ^§^ *P* < 0.05, ^§§^ *P* < 0.01 and ^§§§^ *P* < 0.001 as 200 ng/g BW vs. the saline treated controls.

Specifically aiming at developing the understanding of the chronic impact of CCK8 on Siberian sturgeon food intake, fish were *i*.*p*. injected everyday with three distinct doses of CCK8 (50, 100 and 200 ng/g BW) or saline for 7 days. CCK8 given at 50 ng/g BW significantly reduced food intake only at the day-4 (*P* < 0.05). The doses of CCK8 (100 and 200 ng/g BW) significantly reduced the food intake all through the whole experiment (Fig. 5A), in addition to significantly reducing the cumulative food intake from day-1 to day-7 in comparison with the saline group (Fig. 5B).

### The effects of chronic i.p. injection of CCK8 on the expression of appetite factors

**Figure 6:**
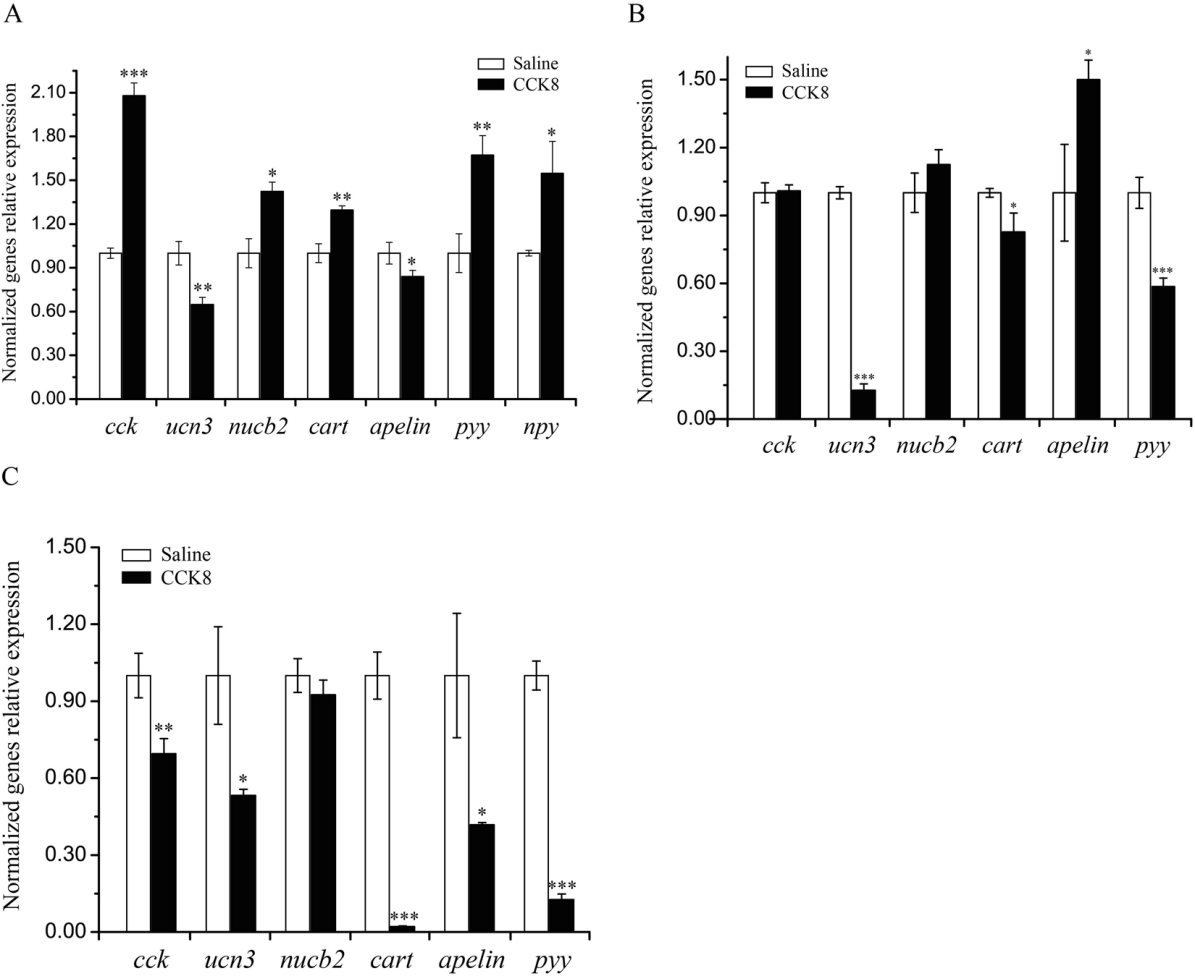
Effects of chronic *i*.*p*. injection of CCK8 (100ng/g BW) for 7 days on the expression of feeding related factors in the whole brain (**A**), stomach (**B**) and duodenum (**C**) in Siberian sturgeon. Asterisks represent significant differences between the two objects (Student’s *t*-test, *P < 0.05, **P < 0.01 and ***P < 0.001).

In this research work, sampling was performed for the fish continuously *i*.*p*. injected with 100 ng/g BW CCK8 or saline for 7 days, together with detecting the expression levels of feeding related factors in the brain, stomach and duodenum. The findings revealed the fact that the mRNA expressions of *cck*, *nucb2*, *cart*, *pyy* and *npy* increased significantly in CCK8 group in comparison with the saline group. On the other hand, the expressions of *ucn3* and *apelin* were significantly decreased in the brain of Siberian sturgeon (Fig. 6A). In stomach, the level of *apelin* exhibited a significant increased, the expression of *ucn3*, *cart* and *pyy* showed significant decreased, and the expression of *cck* and *nucb2* were not considerably changed in CCK8 group in comparison with the control (Fig. 6B). CCK8 induced a substantial decreased in the expressions of *cck*, *ucn3*, *cart*, *apelin* and *pyy* in duodenum; nevertheless, there was observed no significant change in *nucb2* expression (Fig. 6C).

### The effects of i.p. co-injection of CCK8 and CCK receptor antagonists on food intake

**Figure 7:**
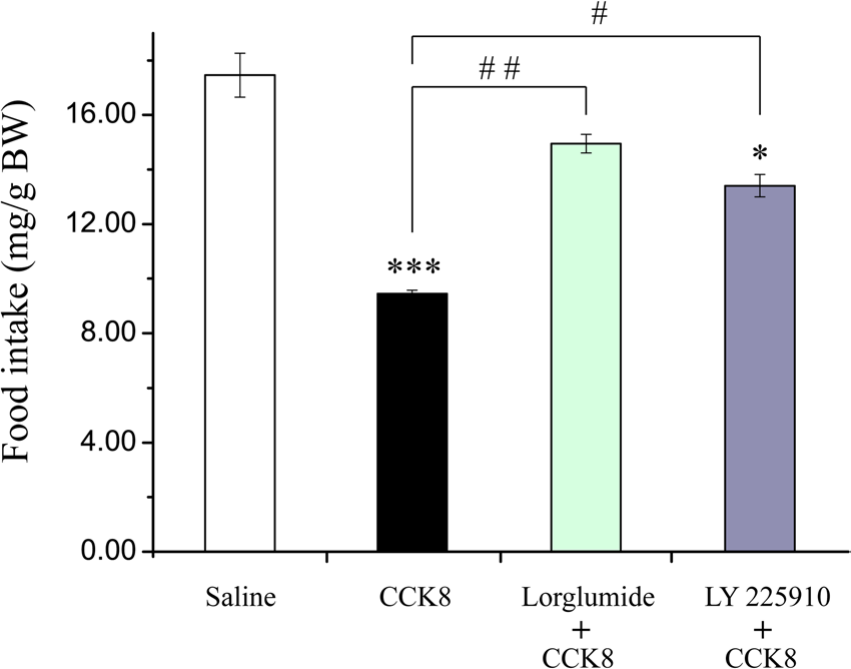
Effects of co-injection of CCK8 and CCK receptor antagonists on 1 h food intake. CCK8 (100ng/g BW), Lorglumide (CCK1R selective antagonist, 1 μg/g BW) and LY 225910 (CCK2R selective antagonist, 0.5 μg/g BW). Asterisks represent comparing with the saline control group (ANOVA, *P < 0.05, **P < 0.01 and ***P < 0.001); Well number represent comparing with the CCK8 group (ANOVA, ^#^ *P* < 0.05, ^##^ *P* < 0.01 and ^###^ *P* < 0.001).

In this study, the co-injection of CCK8 and CCK receptor antagonists were carried out. As shown by the results, CCK8 (100 ng/g BW) *i*.*p*. injection significantly decreased the food intake in 1 h of Siberian sturgeon (Fig. 7). CCK1R selective antagonist, Lorglumide, and CCK8 co-injection delivered an effective safeguard against the inhibitory effect of CCK8 on food intake. Moreover, the food intake was considerably higher than that of CCK8 group, and there was observed no significant difference between Lorglumide and CCK8 co-injection group and the saline group (Fig. 7). CCK2R selective antagonist, LY 225910, also blocked the inhibitory effect of CCK8 on food intake, whereby the food intake was significantly higher than that of CCK8 group, but the food intake of LY 225910 and CCK8 co-injection group still amounted to be considerably lower than that of the saline group (Fig. 7).

### The effects of i.p. co-injection of CCK8 and CCK receptor antagonists on the expression of appetite factors

**Figure 8:**
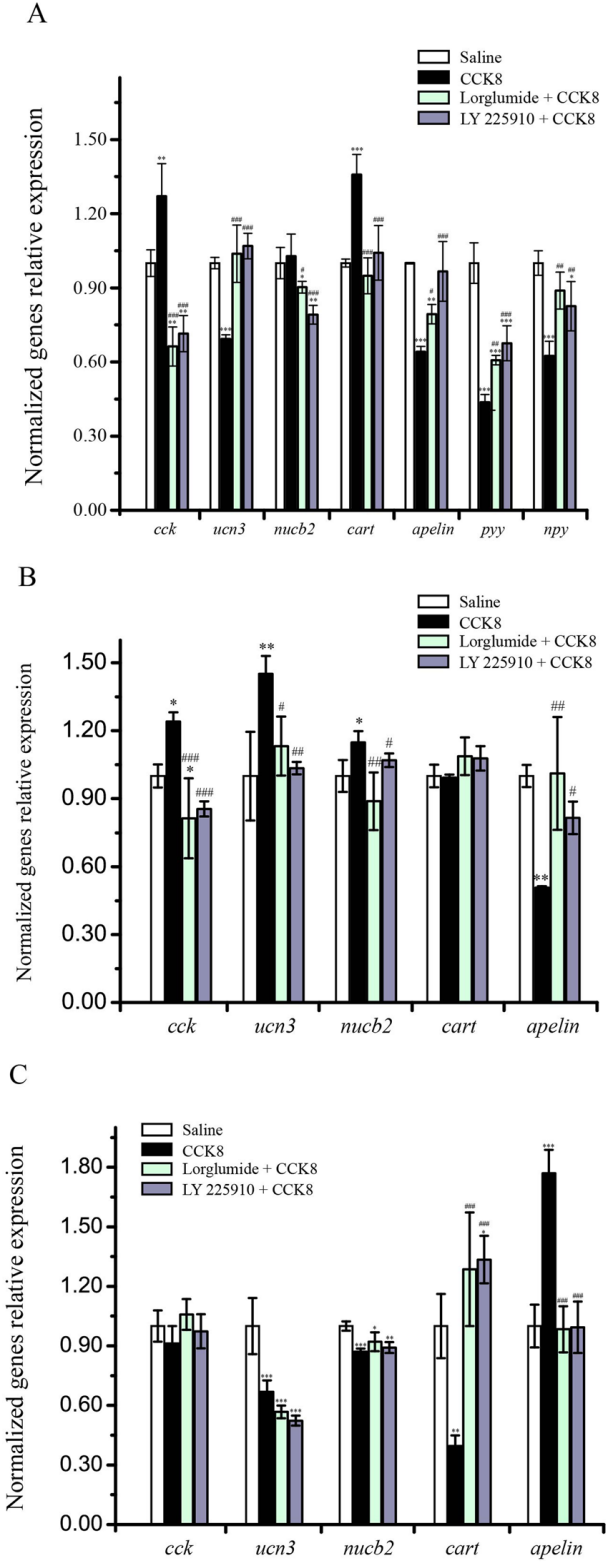
Effects of co-injection of CCK8 and CCK receptor antagonists on the expression of feeding related factors in the whole brain (**A**), stomach (**B**) and duodenum (**C**) in Siberian sturgeon. CCK8 (100ng/g BW), Lorglumide (CCK1R selective antagonist, 1 μg/g BW) and LY 225910 (CCK2R selective antagonist, 0.5 μg/g BW). Asterisks represent comparing with the saline control group (ANOVA, *P < 0.05, **P < 0.01 and ***P < 0.001); Well number represent comparing with the CCK8 group (ANOVA, ^#^ *P* < 0.05, ^##^ *P* < 0.01 and ^###^ *P* < 0.001).

In this research work, the levels of appetite factors in the whole brain, stomach and duodenum were brought under examination to *i*.*p*. co-injection of CCK8 and CCK receptor antagonists. The results attained regarding the brain suggested that CCK8 (100 ng/g BW) induced the expression of *cck* and *cart* mRNA significantly increased. Moreover, the levels of *ucn3*, *apelin*, *pyy* and *npy* were significantly decreased, while the expression of *nucb2* exhibited no apparent change (Fig. 8A). Nevertheless, Lorglumide and CCK8 co-injection resulted into significantly reducing the expressions of *cck*, *nucb2*, *apelin* and *pyy*, while the level of *ucn3*, *cart* and *npy* displayed no substantial changes (Fig. 8A). In addition, LY 225910 and CCK8 co-injection significantly reduced the levels of *cck*, *nucb2*, *pyy* and *npy* mRNA, but *ucn3*, *cart* and *apelin* mRNA levels manifested no sizeable changes (Fig. 8A).

The results in stomach showed CCK8 significantly increased the mRNA expressions of *cck*, *ucn3* and *nucb2*, in addition to significantly reducing the level of *apelin*, but *cart* did not show any significant changes (Fig. 8B). Lorglumide and CCK8 co-injection significantly decreased the expression of *cck*, while the expressions of *ucn3*, *nucb2*, *cart* and *apelin* exhibited no noteworthy changes (Fig. 8B). Moreover, LY 225910 and CCK8 co-injection did not bring forth considerable changes in the expressions of *cck*, *ucn3*, *nucb2*, *cart* and *apelin* (Fig. 8B).

It was revealed by the results in duodenum that there was no significant change in the expression of *cck* mRNA (Fig. 8C). In comparison with the control group, other three groups significantly reduced the expressions of *ucn3* and *nucb2* (Fig. 8C). Additionally, CCK8 significantly decreased the expression of *cart*, together with increasing the expression of *apelin* (Fig. 8C). Moreover, CCK8 as well as its receptor antagonists co-injection significantly inhibited the changes of *cart* and *apelin* which were stimulated by CCK8, while there was observed no sizeable impact on the expressions of *ucn3* and *nucb2* (Fig. 8C).

## Discussion

In the current research work, Siberian sturgeon *cck* cDNA sequence was obtained for the first time. Through the comparison of CCK amino acid sequences in several animals, it was observed that the C-terminal peptide was highly conserved, particularly octapeptide structure. In an interesting manner, the octapeptide structure of Siberian sturgeon CCK displays consistency with that of mammals but different from other fish species, for instance rainbow trout^28^, Japanese flounder^7^ and *Schizothorax prenanti* ^14^. The third amino acid of CCK octapeptide of Siberian sturgeon is methionine (Met, M), which had differences with (Leu, L) of most fish species. The result of phylogenetic analysis exhibited consistency with amino acid sequences alignment. Siberian sturgeon CCK was clustered with the sequences of *Latimeria chalumnae*, *Xenopus laevis*, chicken and mammals having the high bootstrap value, while segregated from other fish species. These findings possess similarities with Siberian sturgeon UCN3^19^ and Apelin^20^, which were most likely because of the differentiation of Sturgeons in 200-250 million years ago (mya), in addition to the specific genome duplication of some fish species appeared in 50-80 mya and 5.6-11.3 mya^29–32^. This study has reported the expression pattern of *cck* in Siberian sturgeon, which is similar to that in human^33^, African clawed forg^34^, goldfish^6^, Japanese flounder^7^, *Schizothorax prenanti* ^14^ and blunt snout bream^15^, that is *cck* mRNA was abundant in brain and digestive tract, suggesting CCK might exert an impact on the regulation of appetite.

As of now, experts own the belief that different nutritional conditions and nourishment approaches are capable of affecting the appearance of appetite factors. For instance, fasting stimulates the presence of starvation components and decreases the level of satiety elements^35^. In order to understand whether CCK of Siberian sturgeon participated in controlling appetite, *periprandial* experiment, fasting experiment and re-feeding experiment were carried out. The result showed that *cck* mRNA levels were significantly increased postprandial in the whole brain and duodenum, which suggest that CCK acts as a satiety factor in Siberian sturgeon. Consistent with our results, the expression of *cck* in the hypothalamus and intestine of *Schizothorax prenanti* were significantly increased subsequent to feeding 1 h and 3 h^14^. In the same manner, it has been highlighted that the expression of *cck* mRNA were significantly increased in brain after feeding in goldfish^36^, Atlantic salmon^37^ and channel catfish^38^. In the current study, the level of *cck* was significantly higher in the whole brain after feeding, while significantly lower in the duodenum. These results suggest that CCK is an anorexia factor that performs a distinct role in the signal transmission rate and duration between central and peripheral system. Furthermore, CCK is likely to perform a function of a short effector in peripheral tissues. Similar to our results, several studies have reported that the expression of *cck* was significantly decreased in the brain or intestine after fasting in Atlantic salmon^9^ and yellowtail^8^. Furthermore, the level of *cck* in brain manifested a considerable decline following fasting for 7 days or 15 days, in addition to significantly increasing after re-feeding returned to the control level that were reported in grass carp^12^, *Schizothorax prenanti* ^14^ and blunt snout bream^15^. That is the reason these results suggest that CCK performs the function of a satiety factor with the long-term role in Siberian sturgeon. On the bases of the findings from feeding experiments, we developed a hypothesis that CCK inhibits the food intake of Siberian sturgeon in both the short-term and long-term. Therefore, acute and chronic *i*.*p*. injection experiments of CCK8 were carried out.

As revealed by the findings of acute experiments, CCK8 (100 and 200 ng/g BW) significantly reduced the food intake within 1 h and the cumulative food intake within 3 h. These results show consistency with the change of *cck* expression after feeding, which indicates that CCK is a satiety factor involved in the short-term feeding regulation in Siberian sturgeon. In the same manner, rat *i*.*p*. injection of CCK8 decreased the food intake in 0.5, 1 and 2 h with a dose-dependent pattern^39^. Lo *et al*. discovered that *i*.*p*. injection of CCK8 significantly brought down the food intake of rat in 2 h when the dose amounted to be 0.25 and 0.5 ug/kg BW^40^. Moreover, it was reported that CCK8 significantly reduced the food intake of chicken in 1.5 h by *i*.*p*. injection^41^. However, the information regarding the effect of CCK8 on food intake in fish species is limited. Penney and Volkoff reported that *i*.*p*. injection of 50 ng/g BW CCK8 significantly inhibited the food intake within 0.5 h in cavefish^23^. Furthermore, it was also discovered that both *i*.*p*. and intraventricular injection of CCK8 inhibited the food intake in goldfish^42^. In addition, in recent years, it has been reported that intraventricular injection of CCK8 or oral CCK8 inhibited the food intake in animals including mice^43^, chicken^41^, goldfish^44^, coho salmon^45^ and European sea bass^46^. These findings display agreement with the results of the current research work indicating CCK8 inhibits the short-term food intake in Siberian sturgeon.

As revealed by the chronic experiment, CCK8 (100 and 200 ng/g BW) significantly reduced the daily food intake as well as cumulative food intake from 1 to 7 days. These results exhibited consistency with the results of fasting and re-feeding experiment, which indicates CCK played a quintessential role in regulating the long-term food intake. To the best of our understanding, it was the first report that chronic *i*.*p*. injection of CCK8 inhibited the food intake in animal. Nevertheless, in European sea bass, it has been reported that oral 0.25 mg/kg BW CCK8 in diets significantly lowered the food intake within 5 days^46^. This report possesses similarity with our research and indicates that chronic treatment with CCK8 can reduce the food intake in fish. Therefore, CCK, as a satiety factor, is involved in both the short-term as well as the long-term feeding regulation in Siberian sturgeon.

Our group has been in long engagement to study the regulation of fish feeding. Recently, there have been identified lots of feeding related factors in Siberian sturgeon including *cck*, *pyy*, *ucn3*, *apelin*, *nucb2*, *cart* and *npy*. So, the expressions of these factors were detected in this study. In view of these facts that *cck* mRNA expression is abundant in the whole brain, and duodenum and stomach is the critical location for both food acquisition and storage, so the whole brain, stomach and duodenum were chosen to determine mRNA expressions of feeding related factors. However, the earlier research works have discovered that the expression of *npy* was abundant in the whole brain while being low in peripheral tissues in Siberian sturgeon (unpublished). This is the reason this study only detected the expression of *npy* in the whole brain. Our results showed that chronic *i*.*p*. injection of CCK affect the expression of appetite factors in the brain, stomach and duodenum. Similarly, it was reported that *i*.*p*. injection of CCK8 significantly reduced the expression of *npy*, while making no impact on the expression of *cart* in the whole brain of cavefish^23^. The dissimilarity with our findings was that the rats *i*.*p*. injected with CCK8 posed no effect on the expressions of *npy* and *cart* in the hypothalamus^39^. Differences in empirical findings are likely to be the different injection doses and the detection tissues or animals. In this study, the expression patterns of feeding related factors were reversed in the whole brain, stomach and duodenum, such as *cart*, *apelin* and *pyy*. On the bases of these findings, we developed a hypothesis that CCK8 regulates the food intake in Siberian sturgeon by different pathways in central system and peripheral tissues. Furthermore, earlier reports have revealed the fact that CCK possesses two kinds of receptors, of which CCK1 receptor (CCK1R) was predominantly expressed in peripheral tissues and CCK2 receptor (CCK2R) was extensively expressed in the central system^47–49^. In this way, we develop speculations that the role of CCK in food intake regulation is likely to play through its two receptors. Hence, the experiments of CCK8 and CCK receptor antagonists co-injection programs were performed.

This study revealed the fact that CCK8 (100 ng/g BW) *i*.*p*. injection significantly decreased the food intake of Siberian sturgeon in 1 h. On the other hand, Lorglumide (CCK1R selective antagonist) and CCK8 co-injection effectively reversed the inhibitory effect of CCK8 on food intake. However, LY 225910 (CCK2R selective antagonist) resulted into partially reversing the inhibitory effect of CCK8 on food intake that amounted to be considerably higher as compared with that in CCK8 group, while being significantly lower than saline group. These findings indicate that CCK is likely to be involved in the regulation of food intake primarily with the help of CCK1R and partially by CCK2R. Similarly, it was reported in mice that CCK1R antagonist MK-329 lead to the inhibition of the effect of CCK8 on feeding, while CCK2R antagonist posed no impact^43^. The information about CCK on feeding regulation in fish species is still limited. Himick and Peter *et al*. reported that the feeding behavior of coho salmon observed a significant promotion subsequent to the CCK signal blocked^50^. In European sea bass, it was also reported that oral CCK8 reduced the food intake, but the effect of CCK8 on inhibitory food intake got obstructed while utilizing CCK nonspecific receptor antagonist proglumide^46^. On the bases of our current discovery together with these previous reports, we develop a proposition that the action of CCK8 in feeding regulation is likely to be via its receptors, mainly through CCK1R and partly through CCK2R.

To understand the mechanism of CCK8 in regulating the food intake through it receptors, we examined the levels of feeding related factors in the whole brain, stomach and duodenum. Our findings showed that CCK exerted influence on the expressions of *cck*, *ucn3*, *cart* and *npy* through CCK1R, in addition to influencing the levels of *ucn3*, *cart* and *apelin* through CCK2R in brain. In the second place, we discovered that CCK8 regulates the expression of *cck*, *ucn3*, *nucb2* and *apelin* with the help of two receptors in stomach. In the long run, CCK8 regulated the levels of *cart* and *apelin* through two receptors, in addition to directly affecting the expressions of *ucn3* and *nucb2* in duodenum. Additionally, CCK8 decreased the level of *npy* by CCK1R and decreased the level of *apelin* through CCK2R in the whole brain. These findings exhibited the similar expression patterns with the chronic injection experiments that are distinct in the expression of feeding related factors in the central system and peripheral tissues. To sum up, blocking CCK1R could effective reversed the changes of the feeding related factors expressions that induced by CCK8, but blocking CCK2R barely resulted into part reversal of these alternatives.

In summary, the cDNA sequence of *cck* in Siberian sturgeon was attained for the first time. The current research work indicates that CCK performs as a satiety factor in both short-term and long-term feeding regulation in Siberian sturgeon. In addition, it was also discovered that the pretreatment of CCK1R selective antagonist (Lorglumide) and CCK2R selective antagonist (LY 225910) could lead to the reversal of changes of *cck*, *ucn3*, *cart*, *apelin* and *npy* expression induced by CCK8 in the brain, stomach and duodenum. These results indicate that CCK inhibits the feeding of Siberian sturgeon primarily through CCK1R and partly through CCK2R. This investigation brings forth a theoretical basis to investigate the mechanism of CCK in feeding regulation of fish species. Further studies are required for the investigation of the correlation between CCK and other feeding related factors for the enrichment of the feeding regulatory network of CCK.

## Electronic supplementary material

Supplementary Information
