# Supplementary material for: CCK reduces the food intake mainly through CCK1R in Siberian sturgeon (Acipenser baerii Brandt)
Source: Sci Rep. 2017 Sep 29;7:12413. doi: 10.1038/s41598-017-12646-3 (PMC5622057; doi:10.1038/s41598-017-12646-3)

CCK reduces the food intake mainly through CCK1R in Siberian sturgeon (*Acipenser baerii* Brandt)

Xin Zhang[1], Ni Tang[1], Jinwen Qi[1], Shuyao Wang, Jin Hao, Yuanbing Wu, Hu Chen, Zhengzhi Tian, Bin Wang, Defang Chen, Zhiqiong Li*

Department of Aquaculture, College of Animal Science and Technology, Sichuan Agricultural University, 211# Huimin Road, Chengdu, China.

*Correspondence and reprint requests to: Prof. Zhiqiong Li.

Xin Zhang, Ni Tang and Jinwen Qi contributed equally to this work.

Department of Aquaculture

College of Animal Science and Technology

Sichuan Agricultural University

Tel: 86-028-86291010

E-mail: lizhiqiong454@163.com

Disclosure summary: The authors have nothing to disclose.

Supplementary Fig. 1 Amino acid sequence alignment of Siberian sturgeon CCK with other vertebrates, including Human (*Homo sapiens*, AH002739), Rat (*Rattus norvegicus*, NM_012829), Chicken (*Gallus gallus*, NM_001001741), Chinese soft-shelled turtle (*Pelodiscus sinensis*, XM_006131816), African clawed frog (*Xenopus laevis*, Z47558/ Z47557), Coelacanth (*Latimeria chalumnae*, XM_006013037), rainbow trout (*Oncorhynchus mykiss*, NM_001124611/ NM_001124345), *Schizothorax prenanti* (KJ194185), *Astyanax mexicanus* (XM_007250428), Nile tilapia (*Oreochromis niloticus*, NM_001279730), Large yellow croaker (*Larimichthys crocea*, KC899121/ KF938690), Atlantic herring (*Clupea harengus*, NM_001309836), Japanese eel (*Anguilla japonica*, AB109556) and Japanese flounder (*Paralichthys olivaceus*, AB009281). The colored amino acids highlight the differences in conservation of the amino acids between species of CCK.

Supplementary Fig. 2 Phylogenetic analysis of CCK amino acid sequences was performed using MEGA 7.0 software of maximum likelihood method with default settings. Scale bar indicates the substitution rate per residue. Numbers at nodes indicate the bootstrap value, as percentages, obtained for 1000 replicates. GenBank accession numbers were listed in the legend of Supplementary Fig.1.

Supplementary Fig. 1

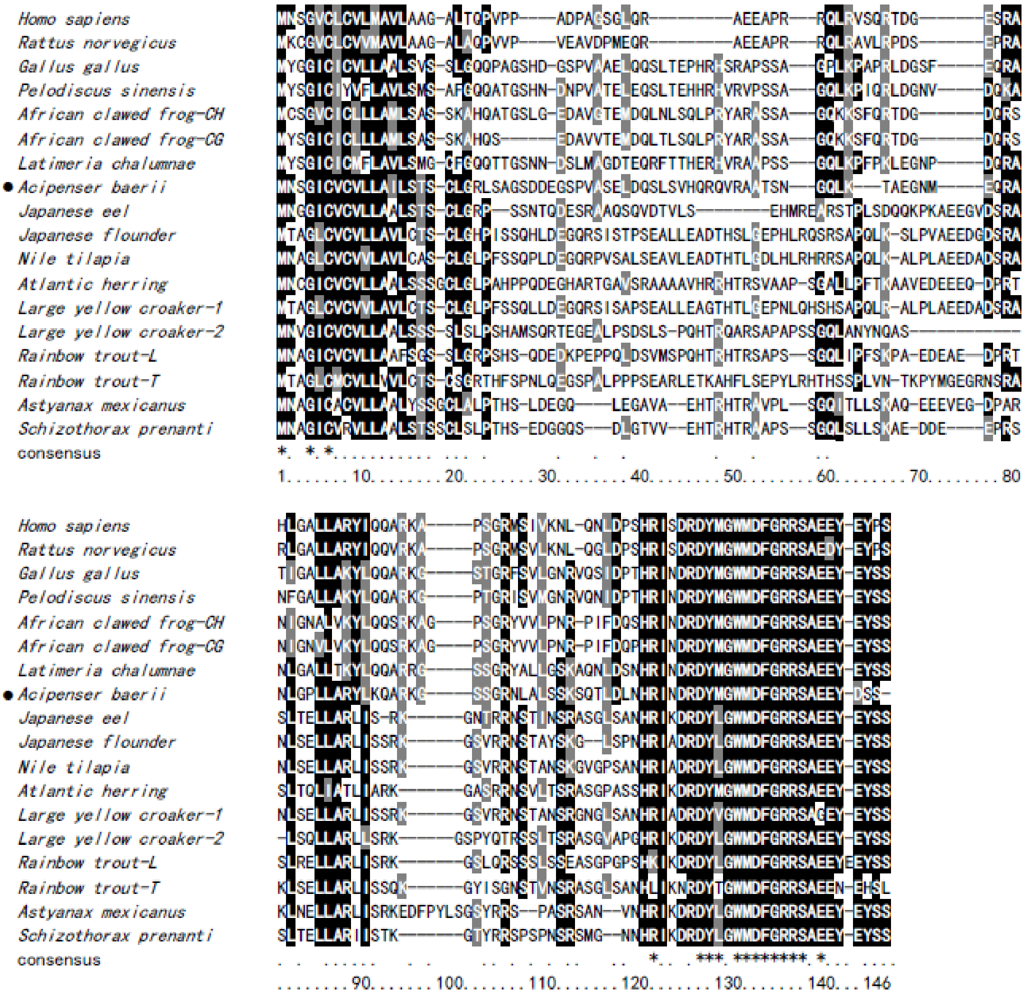

Supplementary Fig. 2

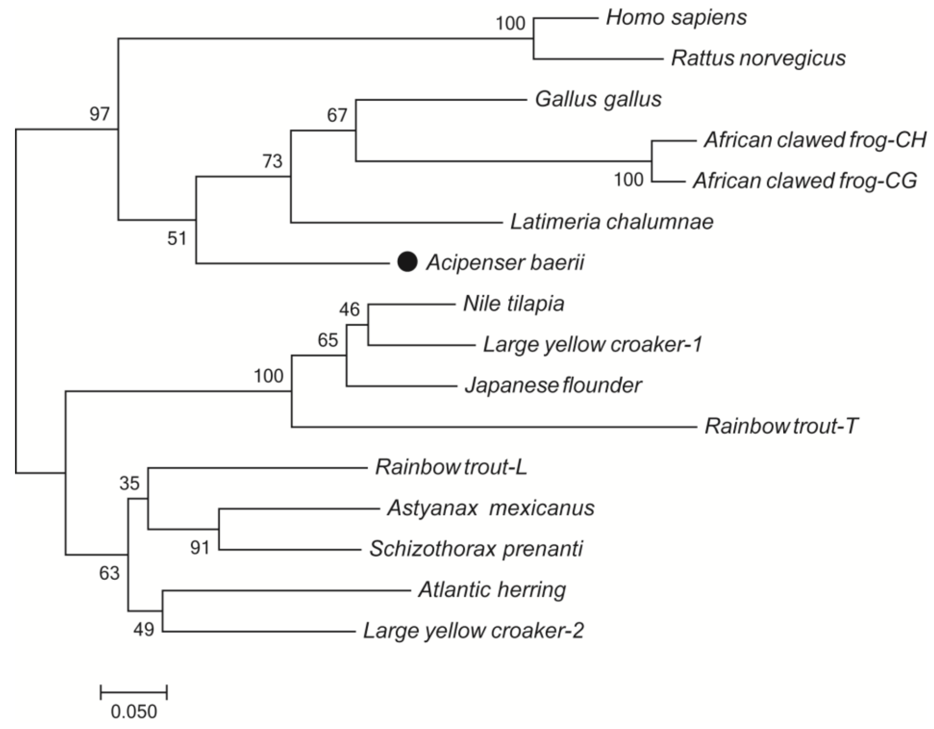

Supplement: Supplementary file 1 — Supplementary Information [file 41598_2017_12646_MOESM1_ESM.pdf]
